# Strategies Towards Antigen-Specific Treatments for Membranous Nephropathy

**DOI:** 10.3389/fimmu.2022.822508

**Published:** 2022-02-03

**Authors:** Sarah M. S. Köllner, Larissa Seifert, Gunther Zahner, Nicola M. Tomas

**Affiliations:** III. Department of Medicine, University Medical Center Hamburg-Eppendorf, Hamburg, Germany

**Keywords:** membranous nephropathy, antigen-specific antibodies, B cells, chimeric autoantibody receptor, sweeping antibody, autoantibodies

## Abstract

Membranous nephropathy (MN) is a rare but potentially severe autoimmune disease and a major cause of nephrotic syndrome in adults. Traditional treatments for patients with MN include steroids with alkylating agents such as cyclophosphamide or calcineurin inhibitors such as cyclosporine, which have an undesirable side effect profile. Newer therapies like rituximab, although superior to cyclosporine in maintaining disease remission, do not only affect pathogenic B or plasma cells, but also inhibit the production of protective antibodies and therefore the ability to fend off foreign organisms and to respond to vaccination. These are undesired effects of general B or plasma cell-targeted treatments. The discovery of several autoantigens in patients with MN offers the great opportunity for more specific treatment approaches. Indeed, such treatments were recently developed for other autoimmune diseases and tested in different preclinical models, and some are about to jump to clinical practice. As such treatments have enormous potential to enhance specificity, efficacy and compatibility also for MN, we will discuss two promising strategies in this perspective: The elimination of pathogenic antibodies through endogenous degradation systems and the depletion of pathogenic B cells through chimeric autoantibody receptor T cells.

## Introduction

Membranous nephropathy (MN) is a rare but potentially severe kidney disease and a major cause of nephrotic syndrome in adults. According to the new KDIGO 2021 Clinical Practice Guidelines, a nephrotic syndrome is defined as proteinuria of more than 3.5 grams per 24 hours or a protein-to-creatinine ratio of more than 3 g/g in combination with low plasma albumin, peripheral edema, and hyperlipidemia (1). The descriptive term “membranous” refers to the prominent change that is classically seen in light microscopy: a diffuse thickening of the glomerular basement membrane (2). Additionally, granular depositions of immunoglobulins and complement components can be detected by immunofluorescence microscopy, suggesting a role of both autoantibodies and the complement system in the pathogenesis of MN. The hallmark findings in electron microscopy include electron-dense deposits in a subepithelial localization, i.e. on the outer aspect of the glomerular basement membrane (GBM), and an extensive effacement of podocyte foot processes. Due to the prominent glomerular IgG positivity in biopsies of affected patients, MN has long been assumed to be an antibody-mediated autoimmune disease. The discoveries of several target antigens for circulating autoantibodies in MN patients in the past decade has corroborated this assumption. These targets include neutral endopeptidase (NEP) (3), M-type phospholipase A2 receptor (PLA2R1) (4), thrombospondin type-1 domain-containing protein 7A (THSD7A) (5), neural epidermal growth factor-like 1 protein (NELL-1) (6), semaphorin 3B (SEMA3B) (7), protocadherin 7 (PCDH7) (8), NCAM1 (9) and HTRA1 (10). PLA2R1-associated MN is diagnosed in around 70% of cases and thus represents the most common MN sub-entity.

**Figure 1:**
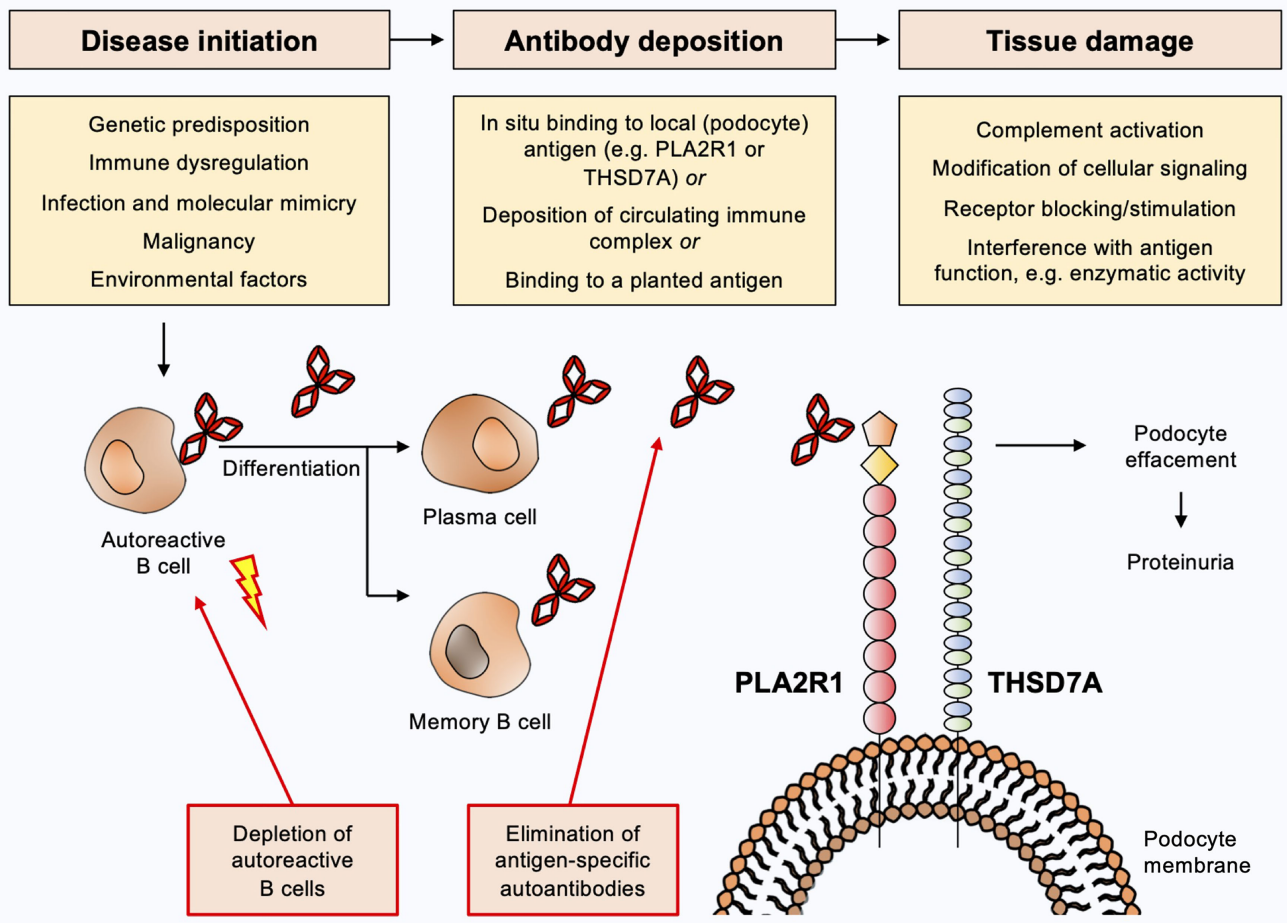
Current view on the pathogenesis of membranous nephropathy and potential targets for antigen-specific treatments. Predisposing factors such as underlying genetic dispositions and/or immune dysregulation in combination with an initiating trigger such as an infection, a malignancy or environmental factors may lead to loss of tolerance for the respective autoantigen with consecutive activation of B cells and production of autoantibodies. B cells differentiate to plasma and/or memory B cells, which in turn produce large amounts of autoantibodies. These autoantibodies reach the kidney *via* the circulation and bind to their target antigen (e.g. PLA2R1 or THSD7A), which induces damage to podocytes with subsequent urinary loss of plasma proteins. Complement-dependent and complement-independent injury mechanisms are likely to be involved. Depletion of autoantibody-producing B cells or elimination of pathogenic autoantibodies constitute potential antigen-specific treatments for MN.

The current view on the pathogenesis of MN (**Figure 1**) is that predisposing factors, such as an underlying genetic disposition and/or immune dysregulation, in combination with an initiating trigger such as an infection, a malignancy or environmental factors lead to loss of tolerance for the respective autoantigen with consecutive activation of B cells and production of autoantibodies (11–18). B cells differentiate towards plasma and/or memory B cells, which in turn produce large amounts of autoantibodies. These autoantibodies reach the kidney *via* the circulation and bind to their target antigen, which is assumed to induce damage to podocytes with subsequent loss of plasma proteins to the urine, likely *via* complement-dependent and complement-independent mechanisms (19). Complement-independent mechanisms have been ill-defined so far, but may include modification of cellular signaling, antigen/receptor blocking or stimulation or interference with the antigens’ biological function, e.g. enzymatic activity. Importantly, the direct pathogenicity of autoantibodies has been demonstrated by transfer experiments of patient-derived anti-NEP and anti-THSD7A autoantibodies, which cause MN in animals (3, 20). Even though such a mechanism has not been demonstrated for anti-PLA2R1 autoantibodies, the association of high autoantibody levels with an unfavorable clinical outcome in patients with PLA2R1-associated MN and the development of MN transgenic mice expressing the murine PLA2R1 after transfer of rabbit anti-PLA2R1 antibodies strongly argue for a pathogenic role of anti-PLA2R1 autoantibodies (21, 22). Alternative proposed mechanisms of immune deposit formation in MN include glomerular deposition of preformed immune complexes and binding of circulating antibodies to a planted antigen (23, 24), but the relevance of these mechanisms is less clear.

## Current Treatments For MN

The clinical outcome in patients with MN is highly variable with about one-third of patients experiencing spontaneous remission within one year after diagnosis, whereas another 20-30% develop end-stage renal disease within 10 years (25). The identification of patients with an unfavorable outcome, which would benefit from early immunosuppressive treatment, represents a major clinical challenge. Classical treatment regimens for MN include the use of steroids in combination with alkylating agents or calcineurin inhibitors (26–29). Recently, two large prospective clinical trials have investigated the use of rituximab in the treatment for MN. The MENTOR trial compared rituximab with cyclosporine and found rituximab to be superior to cyclosporine in maintaining remission for up to 24 months (30). The RI-CYCLO trial found comparable remission rates with the use of rituximab and cyclophosphamide and no significant differences in the safety profiles of these medications (31).

The novel KDIGO guidelines define clusters of patients according to the risk of disease progression and loss of renal function. Categories comprise low risk, moderate risk, high risk, and very high risk, depending on proteinuria, serum albumin, estimated GFR (eGFR), and anti-PLA2R1 antibody levels (1). Patients with a low or moderate risk of disease progression are usually treated with optimal supportive care (e.g. antiproteinuric therapy with renin-angiotensin aldosterone system inhibition (RAAS) and blood pressure control) and monitored for 3-6 months. In case of worsening proteinuria, eGFR or antibody levels, patients should be evaluated for treatment with rituximab or calcineurin inhibitors. Patients with a high risk of disease progression should be treated with rituximab or cyclophosphamide plus steroids or a calcineurin inhibitor plus rituximab and patients with a very high risk should receive cyclophosphamide plus steroids (1).

While calcinerin inhibitors and cyclophosphamide are broad immunosuppressants, rituximab is targeting B cells expressing the surface marker CD20. Initially developed as a lymphoma treatment, it acts through different mechanisms of action: i) Antibody-dependent cellular cytotoxicity (ADCC) through either NK cells, monocytes or granulocytes (32–34) (ii) apoptosis of B cells through caspase 3 activation (35) and iii) complement-dependent cytotoxicity (36, 37).

In summary, the targets of autoimmunity in MN have been increasingly understood and therapies have shifted from broad immunosuppression using alkylating agents and calcineurin inhibitors towards a more pathogenesis-based treatment targeting autoantibody-producing B cells using rituximab. However, rituximab treatment may entail resistance towards apoptosis, ADCC, CDC and downregulation or loss of CD20 (38), which can decrease treatment efficacy. It also affects pre-existing protective antibodies, reduces the body’s ability to generate an immune response against foreign organisms, and greatly decreases the response to vaccination. These are undesired effects of B cell–targeted treatments, in particular in the setting of a worldwide pandemic (39), and may limit the use of agents such as rituximab in the treatment of antibody-mediated diseases. Additionally, the increasing knowledge on specific antigens in MN principally enables antigen-specific treatment strategies, which would ideally target the immunological disease mechanisms while sparing protective immunity. Such antigen-specific therapies would have enormous potential to enhance specificity, efficacy and compatibility. Hence, there is a considerable gap between the increasing knowledge on the pathogenic role of autoantibodies and autoantigens in MN on the one side and the currently available treatments with limited specificity on the other side.

In the following, we will discuss two promising strategies that could be applied in the field of MN: The elimination of pathogenic antibodies through endogenous degradation systems and the elimination of autoreactive B cells through chimeric autoantibody receptor T cells (**Figure 1**).

## No Sweeping Under The Rug: The Sweeping Antibody Technology

Physiologically, immunoglobulins (IgG) are constantly taken up by endothelial cells and shuttled to the sorting endosome. At pH 6 inside the sorting endosome IgG binds to the neonatal Fc receptor (FcRn) and is transported to the cell surface. Now at pH 7.4 the IgG is released from the FcRn back into the circulation due to a reduced affinity at neutral pH. This system prolongs the half-life of IgG in the circulation to approximately 30 days (40). Consequently, antibodies may also prolong the half-life of their target antigen by constant recirculation from endothelial cells back to the plasma.

Sweeping antibodies as potential therapeutics to remove soluble antigens from plasma were first described by Igawa et al. (41). Sweeping antibodies are engineered IgG that remove soluble antigens from the circulation, which is enabled by two distinct modifications: a pH-dependent binding to the antigen and an increased affinity of the antibody Fc part to an Fc receptor (FcR) (41). A pH-dependent antigen binding can be achieved by histidine mutagenesis, meaning that amino acids in the Fab region of the antibody are substituted for histidines. In the acidic environment of the sorting endosome, the protonated histidines lead to conformational changes in the Fab region of the sweeping antibody, which results in weakening of the binding to the antigen and finally dissociation of antibody and antigen. Enhanced immune complex uptake into the endosome is achieved by mutations in the Fc region, increasing the binding to either FcRn or FcγRIIB.

After injection, a sweeping antibody binds its target molecule in the circulation. The resulting immune complex is then taken up into endothelial cells by pinocytosis or FcR-mediated endocytosis. In the sorting endosome, the pH shifts from neutral to slightly acidic (pH 6) and the antigen is released from the sweeping antibody. While the sweeping antibody – bound by the FcRn – is shuttled to the cell surface and released there, the antigen is degraded in the lysosome. The enhanced affinity to the FcR allows efficient recirculation of the therapeutic sweeping antibody from endothelial cells to the plasma, which in turn leads to further removal of antigens from the circulation, creating the “sweeping” effect. Several different mutations have been described that enhance active internalization of antigen-antibody complexes *via* FcRn, leading to efficient degradation of the antigen (42–44).

Sweeping can also occur through the FcγRIIB (45). FcγRIIB is the only inhibitory Fc receptor with an immunoreceptor-based inhibitory motif (ITIM) and normally has a very low affinity for IgG monomers (IgG1<IgG2a=IgG2b<IgG3) (46). FcγRIIB is, for example, expressed on B cells regulating their activation (47), and on myeloid-derived cells modulating endocytosis through clathrin-coated pits (48). Liver sinusoidal endothelial cells (LSECs) are scavenger cells specialized to clear blood from smaller immune complexes through pinocytosis (49). In mice, three quarters of all FcγRIIB are expressed in the liver and 90% of liver FcγRIIB is found on the surface of LSECs (50). Interestingly, Ganesan *et al*. could demonstrate that small immune complexes are cleared *via* FcγRIIB-mediated uptake into the liver (50). Binding to FcγRIIB can be dramatically enhanced by mutating the Fc part of the sweeping antibody, leading to efficient clearing of soluble antigens (51).

Recently, it could be demonstrated that the sweeping antibody principle can also be applied for the elimination of antigen-specific antibodies. Devanaboyina et al. (52) used therapeutic antibodies including fragments of either the myelin oligodendrocyte glycoprotein (MOG), an antigen in multiple sclerosis, or HER2, a tumor protein (52). The therapeutic antibodies specifically bound to anti-MOG and anti-HER2 antibodies, which lead to rapid clearance of the resulting immune complex in mice. In a follow-up study, the authors could demonstrate a therapeutic effect using similar constructs in a mouse model of multiple sclerosis (53).

**Figure 2:**
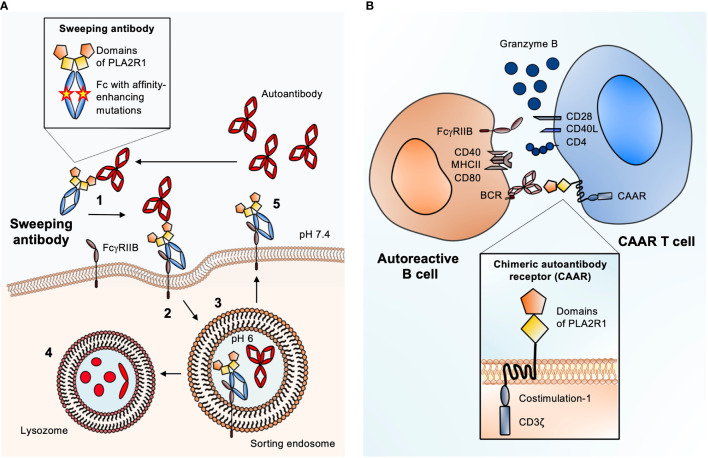
Antigen-specific therapies suited for MN. **(A)** Schematic of the sweeping antibody principle with enhanced FcγRIIB binding. 1. After injection, sweeping antibody and autoantibody bind in the circulation. 2. Scavenger cells like liver sinusoidal endothelial cells (LSECs) that express FcγRIIB bind the circulating immune complex (IC) and internalize it through pinocytosis. 3. Due to a pH shift from neutral to pH 6 inside the sorting endosome, the autoantibody is released from the IC-receptor complex. 4. The autoantibody is degraded inside the lysosome. 5. The Fc receptor-bound sweeping antibody is returned to the surface and can bind new circulating autoantibodies causing the “sweeping” effect. Magnified: structure of a PLA2R1 sweeping antibody: The Fab part is substituted for the most N-terminal domains of PLA2R1 (cysteine-rich and fibronectin type II) to create specificity for anti-PLA2R1 autoantibodies. Mutations in the Fc part of the sweeping antibody enhance the affinity towards FcγRIIB. **(B)** Schematic of chimeric autoantibody receptor (CAAR) T cell principle. The CAAR comprises fragments of the target antigen (in this case the cysteine-rich and fibronectin type II domains of PLA2R1), a transmembrane domain, and several intracellular signaling domains. The CAAR enables binding to a B cell, which expresses the corresponding B cell receptor (BCR), a membrane-anchored IgG corresponding to the autoantibody that is produced by the B cell. CAAR-mediated binding of the T cell to the pathogenic B cell leads to release of granzyme B, which eliminates the target B cell. Magnified: structure of a second generation CAAR. It includes the autoantibody receptor as a ectodomain, here the most N-terminal domains of PLA2R, a transmembrane part and an endodomain with co-stimulation module and the CD3ζ with three immunoreceptor tyrosine-based activation motifs (ITAMs). The co-stimulatory domain improves the half-life *in vivo*, proliferation and cytotoxity of the CAAR T cell.

The increasing knowledge on pathogenic autoantibodies in MN and their target antigens offers excellent conditions to apply the sweeping antibody concept also in this disease. Sweeping antibodies with the Fab region substituted for an antigen fragment could be generated and tested for their capacity to bind and eliminate specific autoantibodies. As virtually 100% of patients with PLA2R1-associated MN have antibodies against the most N-terminal domain (the cysteine-rich domain) of PLA2R1 (54–56), a sweeping antibody containing this part of PLA2R1 may be a promising candidate – yet therapeutic antibodies could be engineered for any MN antigen and tailored to the immune and epitope profile of each individual patient. After administration, the therapeutic sweeping antibodies bind their targets, the pathogenic autoantibodies, in the circulation. The resulting immune complex is then taken up by liver LSECs through enhanced affinity for FcγRIIB or by other endothelial cells *via* FcRn, resulting in lysosomal degradation of the pathogenic autoantibodies and potentially recirculation of the therapeutic sweeping antibody (**Figure 2A**). Fewer autoantibodies could bind their target antigen in the kidney, potentially leading to amelioration of disease activity. This can improve the course of MN without affecting parts of the immune system that are essential for the body’s defense e.g. against infection. In summary, the elimination of pathogenic autoantibodies through endogenous degradation systems represents a promising therapeutic strategy for patients with MN.

## Striking The Evil At Its Roots: Chimeric Autoantibody Receptor T Cells

Chimeric antigen receptor (CAR) T cells are a promising treatment for cancer, used with remarkable success for example in refractory or relapsed B cell lymphoma (57–59). To obtain such CAR T cells, peripheral blood mononuclear cells from the patient’s blood are isolated and stimulated with interleukin 2 and anti-CD3 antibodies, leading to their proliferation. The T cells are transduced with a construct encoding for the CAR of interest, e.g. the antigen-binding domain of an anti-CD19 antibody fused to a transmembrane and several intracellular signaling domains such as 4-1BB and CD3ζ (60). Upon transfusion back to the patient, the CAR T cells eliminate cells expressing the target antigen, in this case CD19, which is a marker of B cells. A huge advantage of this treatment is the generation of long-term memory CAR T cells, which offer the opportunity for constant elimination of newly emerging target cells without the need for repetitive dosing.

This strategy can also be applied for the treatment of antibody-mediated autoimmune diseases, with one essential modification: the antigen-binding domain of a conventional CAR is replaced by a part of the autoantigen of interest, resulting in a chimeric autoantibody receptor (CAAR). A T cell expressing this CAAR (CAAR T cell) will bind to and eliminate B cells that express the corresponding B cell receptor (BCR), a membrane-bound immunoglobulin matching the antibody produced by this particular cell clone. This approach was firstly tested in an animal model of pemphigus vulgaris (PV). T cells expressing a CAAR consisting of the PV autoantigen desmoglein 3 fused to CD137-CD3ζ specifically eliminated anti-desmoglein 3-specific autoreactive B cells (61). Moreover, the CAAR T cells erased their targets even in the presence of circulating anti-desmoglein 3 antibodies and did not show significant off target effects. In a follow-up study, the authors preclinically examined the pharmacodynamics and toxicity of this CAAR and showed that the desmoglein 3-CAAR T cells specifically deplete primary human desmoglein 3-specific B cells from PV patients and is effective in an active animal model of PV (62).

Applying the CAAR T cell strategy for the treatment of MN would be a pioneering approach. The antibody binding sites in the antigens PLA2R1 and THSD7A have been already characterized, in parts even down to the level of single antigen domains and smaller epitopes (54–56, 63–65). Given this knowledge, to achieve an optimal intermembrane distance of the immunologic synapse (the space between the CAAR T cell and the target B cell) and minimize potential off-target effects, it appears reasonable to fuse smaller fragments of PLA2R1 or the other MN antigens to the chimeric receptor (**Figure 2B**). In case of PLA2R1 and THSD7A the most N-terminal regions are considered as immunological hot spots, as most reactivity with patient autoantibodies is found in these areas (56, 63, 64). Therefore, a CAAR containing only the N-terminal region of the respective autoantigen might be sufficient to eliminate a number of autoreactive B cells large enough to ameliorate disease. It would also be possible to perform an antibody mapping first, e.g. using domain-specific ELISAs, and tailor the CAAR strategy to the individual epitope profile.

Although the CAR T cell therapy has revolutionized the treatment of malignancies, it has several potential flaws. First, the manufacturing of personalized CAR T cells is very complex, time-consuming and expensive. Second, CAR T cell therapy could involve serious adverse effects, such as the cytokine release syndrome (CRS), caused by the activation of CAR T cells and their production of proinflammatory cytokines (66, 67), or neurotoxicity, which often accompanies CRS, possibly due to cerebral endothelial dysfunction (68). Third, it is questionable whether the generation of memory T cells is always desirable in light of many patients achieving full remission of disease after some time.

The generation of CAR NK cells represents an alternative strategy to overcome some of these undesired effects. Due to their limited lifespan, CAR NK cells show a relatively low toxicity towards normal tissue of the recipient (69). They offer the possibility of “off the shelf” therapy as they can be obtained from peripheral blood mononuclear cells of healthy donors, umbilical cord blood, induced pluripotent stem cells, and even NK92 cell lines (70). Currently, most of the CAR NK constructs derive from CAR T cell approaches, but there are attempts to design also CARs tailored specifically for NK cells (71). To date, CAR NK cell therapy seems to be a safe additional approach for cancer therapy, with several clinical trials running (72, 73). In conclusion, CAAR T cell therapy and, alternatively, the safer and likely more cost-efficient and easier to manufacture CAAR NK cell therapy represent an elegant and promising therapeutic strategy for patients with MN.

Notably, is also possible to approach T cell therapy from the opposite site, by regaining immune tolerance. To achieve this, the function of one key player of immune tolerance has to be modified: the so called regulatory T cells or Tregs. About 5-7% of all CD4+ T cells in the human body are Tregs. The identity and function of Tregs are characterized by the expression of various markers such as the cytotoxic T lymphocyte-associated protein-4 (CTLA-4), the interleukin 2 receptor subunit α (CD25), the transcription-maintaining factor STAT5, and the forkhead box P3 (FOXP3). FOXP3 has a key function for the maintenance of immune tolerance (74).

Tregs can be transduced with so called BARs (chimeric B cell-targeting antibody receptors) containing an extracellular domain consisting of the immunodominant parts of the antigen, which means they are basically comparable to CAARs on cytotoxic T cells. Instead of killing the B- cell upon binding, such BAR Tregs suppress antigen-specific B cells directly without affecting the T cell response (75).

## Conclusion

MN is an antibody-mediated autoimmune disease and several autoantigens have been identified over the past years. The direct pathogenicity of the involved autoantibodies has either been shown or is strongly suspected (20, 22), and treatment strategies have shifted towards targeting of B cells (30, 31, 76). In light of these developments, the establishment of antigen-specific treatments represents the consequential continuation of MN therapy. We are aware that the therapeutic concepts discussed in this article have not been tested in MN, not even at an experimental level. However, future research should take into account such potential strategies, particularly as antigen-specific animal models become more and more available (22, 77). Clearly, the possible adverse effects of such treatments should not be overlooked, but there is a realistic chance that they would not be as severe as the ones of currently used therapeutics. Especially in case of CAR T cells, huge efforts are made to reduce side effects and make them more controllable, e.g. through transient transduction, suicide genes or elimination markers (78).

The actual charm of the two treatment approaches that we presented here is the option to combine and apply them as a therapeutic package tailored for the individual patient. As an example, one could imagine the following procedure: After confirming the diagnosis of PLA2R1-associated MN by kidney biopsy, the antibody profile is analyzed using a domain-specific ELISA, revealing reactivity with the cysteine-rich domain. Subsequently, sweeping antibodies containing the cysteine-rich domain are applied to eliminate the circulating autoantibodies. This acutely reduces binding of pathogenic antibodies at the glomerular filtration barrier and additionally paves the way for CAAR T or NK cells carrying a CAAR which contains the cysteine-rich domain. The CAAR T or NK cells thus eliminate autoreactive B cells, which prevents further production of pathogenic antibodies. Without previous clearance of antibodies, CAAR cells may in fact be neutralized by the circulating antibodies binding to the CAAR, potentially making them ineffective in eliminating autoreactive B cells. As an alternative or additive strategy, PLA2R1-specific immune tolerance could be restored by suppression of autoreactive B cells using Tregs expressing a BAR that contains the cysteine-rich domain.

In summary, the increasing knowledge about the targets of autoimmunity in MN offers a huge potential for the application of antigen-specific treatment strategies, which would ideally spare protective immunity.

## Author Contributions

SK and LS contributed equally to this review. NT conceptualized the work. All authors discussed the concept and revised the manuscript. All authors approved the final version of the manuscript.

## Funding

This work was funded by the Deutsche Forschungsgemeinschaft as part of the Emmy Noether Programme “Molecular Mechanisms of Membranous Nephropathy” (TO10-13) to NT and as part of the Sonderforschungsbereich 1192 to NT (project B2). This work was additionally supported by the Deutsche Forschungsgemeinschaft (DFG) as part of the grant ZA163/12-1 to GZ.

## Conflict of Interest

The authors declare that the research was conducted in the absence of any commercial or financial relationships that could be construed as a potential conflict of interest.

## Publisher’s Note

All claims expressed in this article are solely those of the authors and do not necessarily represent those of their affiliated organizations, or those of the publisher, the editors and the reviewers. Any product that may be evaluated in this article, or claim that may be made by its manufacturer, is not guaranteed or endorsed by the publisher.

## References

[1] Kidney Disease: Improving Global Outcomes Glomerular Diseases Work Group. KDIGO 2021 Clinical Practice Guideline for the Management of Glomerular Diseases. Kidney Int (2021) 100(4S):S1–276. doi: 10.1016/j.kint.2021.05.021 34556256

[2] Jones DB. Nephrotic Glomerulonephritis. Am J Pathol (1957) 33(2):313–29. PMC1934622 13402889

[3] Debiec H Guigonis V Mougenot B Decobert F Haymann JP Bensman A. Antenatal Membranous Glomerulonephritis Due to Anti-Neutral Endopeptidase Antibodies. N Engl J Med (2002) 346(26):2053–60. doi: 10.1056/NEJMoa012895 12087141

[4] Beck LH Jr Bonegio RG Lambeau G Beck DM Powell DW Cummins TD. M-Type Phospholipase A2 Receptor as Target Antigen in Idiopathic Membranous Nephropathy. N Engl J Med (2009) 361(1):11–21. doi: 10.1056/NEJMoa0810457 19571279 PMC2762083

[5] Tomas NM Beck LH Jr Meyer-Schwesinger C Seitz-Polski B Ma H Zahner G. Thrombospondin Type-1 Domain-Containing 7A in Idiopathic Membranous Nephropathy. N Engl J Med (2014) 371(24):2277–87. doi: 10.1056/NEJMoa1409354 PMC4278759 25394321

[6] Sethi S Debiec H Madden B Charlesworth MC Morelle J Gross L. Neural Epidermal Growth Factor-Like 1protein (NELL-1) Associated Membranous Nephropathy. Kidney Int (2020) 97(1):163–74. doi: 10.1016/j.kint.2019.09.014 31901340

[7] Sethi S Debiec H Madden B Vivarelli M Charlesworth MC Ravindran A. Semaphorin 3B-Associated Membranous Nephropathy is a Distinct Type of Disease Predominantly Present in Pediatric Patients. Kidney Int (2020) 98(5):1253–64. doi: 10.1016/j.kint.2020.05.030 32534052

[8] Sethi S Madden B Debiec H Morelle J Charlesworth MC Gross L. Protocadherin 7-Associated Membranous Nephropathy. J Am Soc Nephrol (2021) 32(5):1249–61. doi: 10.1681/ASN.2020081165 PMC8259689 33833079

[9] Caza TN Hassen SI Kuperman M Sharma SG Dvanajscak Z Arthur J. Neural Cell Adhesion Molecule 1 is a Novel Autoantigen in Membranous Lupus Nephritis. Kidney Int (2021) 100(1):171–81. doi: 10.1016/j.kint.2020.09.016 PMC8032825 33045259

[10] Al-Rabadi LF Caza T Trivin-Avillach C Rodan AR Andeen N Hayashi N. Serine Protease HTRA1 as a Novel Target Antigen in Primary Membranous Nephropathy. J Am Soc Nephrol (2021) 32(7):1666–81. doi: 10.1681/ASN.2020101395 PMC8425645 33952630

[11] Stanescu HC Arcos-Burgos M Medlar A Bockenhauer D Kottgen A Dragomirescu L. Risk HLA-DQA1 and PLA(2)R1 Alleles in Idiopathic Membranous Nephropathy. N Engl J Med (2011) 364(7):616–26. doi: 10.1056/NEJMoa1009742 21323541

[12] Xu X Wang G Chen N Lu T Nie S Xu G. Long-Term Exposure to Air Pollution and Increased Risk of Membranous Nephropathy in China. J Am Soc Nephrol (2016) 27(12):3739–46. doi: 10.1681/ASN.2016010093 PMC5118492 27365535

[13] Hoxha E Wiech T Stahl PR Zahner G Tomas NM Meyer-Schwesinger C. A Mechanism for Cancer-Associated Membranous Nephropathy. N Engl J Med (2016) 374(20):1995–6. doi: 10.1056/NEJMc1511702 27192690

[14] Hoxha E Beck LH Jr Wiech T Tomas NM Probst C Mindorf S. An Indirect Immunofluorescence Method Facilitates Detection of Thrombospondin Type 1 Domain-Containing 7a-Specific Antibodies in Membranous Nephropathy. J Am Soc Nephrol (2017) 28(2):520–31. doi: 10.1681/ASN.2016010050 PMC5280014 27436855

[15] Stahl PR Hoxha E Wiech T Schroder C Simon R Stahl RA. THSD7A Expression in Human Cancer. Genes Chromosomes Cancer (2017) 56(4):314–27. doi: 10.1002/gcc.22440 28035718

[16] Cremoni M Brglez V Perez S Decoupigny F Zorzi K Andreani M. Th17-Immune Response in Patients With Membranous Nephropathy Is Associated With Thrombosis and Relapses. Front Immunol (2020) 11:574997. doi: 10.3389/fimmu.2020.574997 33324398 PMC7725714

[17] Motavalli R Etemadi J Soltani-Zangbar MS Ardalan MR Kahroba H Roshangar L. Altered Th17/Treg Ratio as a Possible Mechanism in Pathogenesis of Idiopathic Membranous Nephropathy. Cytokine (2021) 141:155452. doi: 10.1016/j.cyto.2021.155452 33571932

[18] Rosenzwajg M Languille E Debiec H Hygino J Dahan K Simon T. B- and T-Cell Subpopulations in Patients With Severe Idiopathic Membranous Nephropathy may Predict an Early Response to Rituximab. Kidney Int (2017) 92(1):227–37. doi: 10.1016/j.kint.2017.01.012 28318628

[19] Tomas NM Huber TB Hoxha E. Perspectives in Membranous Nephropathy. Cell Tissue Res (2021) 385(2):405–22. doi: 10.1007/s00441-021-03429-4 PMC8523383 33825066

[20] Tomas NM Hoxha E Reinicke AT Fester L Helmchen U Gerth J. Autoantibodies Against Thrombospondin Type 1 Domain-Containing 7A Induce Membranous Nephropathy. J Clin Invest (2016) 126(7):2519–32. doi: 10.1172/JCI85265 PMC4922694 27214550

[21] Hoxha E Thiele I Zahner G Panzer U Harendza S Stahl RA. Phospholipase A2 Receptor Autoantibodies and Clinical Outcome in Patients With Primary Membranous Nephropathy. J Am Soc Nephrol (2014) 25(6):1357–66. doi: 10.1681/ASN.2013040430 PMC4033365 24610926

[22] Meyer-Schwesinger C Tomas NM Dehde S Seifert L Hermans-Borgmeyer I Wiech T. A Novel Mouse Model of Phospholipase A2 Receptor 1-Associated Membranous Nephropathy Mimics Podocyte Injury in Patients. Kidney Int (2020) 97(5):913–9. doi: 10.1016/j.kint.2019.10.022 32033781

[23] Debiec H Lefeu F Kemper MJ Niaudet P Deschenes G Remuzzi G. Early-Childhood Membranous Nephropathy Due to Cationic Bovine Serum Albumin. N Engl J Med (2011) 364(22):2101–10. doi: 10.1056/NEJMoa1013792 21631322

[24] Ronco P Debiec H. Pathophysiological Advances in Membranous Nephropathy: Time for a Shift in Patient's Care. Lancet (9981) 2015:1983–92:385. doi: 10.1016/S0140-6736(15)60731-0 26090644

[25] Cattran D. Management of Membranous Nephropathy: When and What for Treatment. J Am Soc Nephrol (2005) 16(5):1188–94. doi: 10.1681/ASN.2005010028 15800117

[26] Ponticelli C Zucchelli P Imbasciati E Cagnoli L Pozzi C Passerini P. Controlled Trial of Methylprednisolone and Chlorambucil in Idiopathic Membranous Nephropathy. N Engl J Med (1984) 310(15):946–50. doi: 10.1056/NEJM198404123101503 6366560

[27] Ponticelli C Zucchelli P Passerini P Cesana B Locatelli F Pasquali S. A 10-Year Follow-Up of a Randomized Study With Methylprednisolone and Chlorambucil in Membranous Nephropathy. Kidney Int (1995) 48(5):1600–4. doi: 10.1038/ki.1995.453 8544420

[28] Cattran DC Greenwood C Ritchie S Bernstein K Churchill DN Clark WF. A Controlled Trial of Cyclosporine in Patients With Progressive Membranous Nephropathy. Canadian Glomerulonephritis Study Group. Kidney Int (1995) 47(4):1130–5. doi: 10.1038/ki.1995.161 7783410

[29] Cattran DC Appel GB Hebert LA Hunsicker LG Pohl MA Hoy WE. Cyclosporine in Patients With Steroid-Resistant Membranous Nephropathy: A Randomized Trial. Kidney Int (2001) 59(4):1484–90. doi: 10.1046/j.1523-1755.2001.0590041484.x 11260412

[30] Fervenza FC Appel GB Barbour SJ Rovin BH Lafayette RA Aslam N. Rituximab or Cyclosporine in the Treatment of Membranous Nephropathy. N Engl J Med (2019) 381(1):36–46. doi: 10.1056/NEJMoa1814427 31269364

[31] Scolari F Delbarba E Santoro D Gesualdo L Pani A Dallera N. Rituximab or Cyclophosphamide in the Treatment of Membranous Nephropathy: The RI-CYCLO Randomized Trial. J Am Soc Nephrol (2021) 32(4):972–82. doi: 10.1681/ASN.2020071091 PMC8017548 33649098

[32] Koene HR Kleijer M Algra J Roos D von dem Borne AE de Haas M. Fc gammaRIIIa-158v/F Polymorphism Influences the Binding of IgG by Natural Killer Cell Fc gammaRIIIa, Independently of the Fc gammaRIIIa-48l/R/H Phenotype. Blood (1997) 90(3):1109–14. doi: 10.1182/blood.V90.3.1109 9242542

[33] Wu J Edberg JC Redecha PB Bansal V Guyre PM Coleman K. A Novel Polymorphism of FcgammaRIIIa (CD16) Alters Receptor Function and Predisposes to Autoimmune Disease. J Clin Invest (1997) 100(5):1059–70. doi: 10.1172/JCI119616 PMC508280 9276722

[34] Clynes RA Towers TL Presta LG Ravetch JV. Inhibitory Fc Receptors Modulate In Vivo Cytotoxicity Against Tumor Targets. Nat Med (2000) 6(4):443–6. doi: 10.1038/74704 10742152

[35] Byrd JC Kitada S Flinn IW Aron JL Pearson M Lucas D. The Mechanism of Tumor Cell Clearance by Rituximab In Vivo in Patients With B-Cell Chronic Lymphocytic Leukemia: Evidence of Caspase Activation and Apoptosis Induction. Blood (2002) 99(3):1038–43. doi: 10.1182/blood.V99.3.1038 11807010

[36] Kumar A Planchais C Fronzes R Mouquet H Reyes N. Binding Mechanisms of Therapeutic Antibodies to Human CD20. Science (2020) 369(6505):793–9. doi: 10.1126/science.abb8008 32792392

[37] Rouge L Chiang N Steffek M Kugel C Croll TI Tam C. Structure of CD20 in Complex With the Therapeutic Monoclonal Antibody Rituximab. Science (2020) 367(6483):1224–30. doi: 10.1126/science.aaz9356 32079680

[38] Rezvani AR Maloney DG. Rituximab Resistance. Best Pract Res Clin Haematol (2011) 24(2):203–16. doi: 10.1016/j.beha.2011.02.009 PMC3113665 21658619

[39] Mehta P Porter JC Chambers RC Isenberg DA Reddy V. B-Cell Depletion With Rituximab in the COVID-19 Pandemic: Where do We Stand? Lancet Rheumatol (2020) 2(10):e589–90. doi: 10.1016/S2665-9913(20)30270-8 PMC7836835 33521659

[40] Sand KM Bern M Nilsen J Noordzij HT Sandlie I Andersen JT. Unraveling the Interaction Between FcRn and Albumin: Opportunities for Design of Albumin-Based Therapeutics. Front Immunol (2014) 5:682. doi: 10.3389/fimmu.2014.00682 25674083 PMC4306297

[41] Igawa T Maeda A Haraya K Tachibana T Iwayanagi Y Mimoto F. Engineered Monoclonal Antibody With Novel Antigen-Sweeping Activity In Vivo. PloS One (2013) 8(5):e63236. doi: 10.1371/journal.pone.0063236 23667591 PMC3646756

[42] Vaccaro C Zhou J Ober RJ Ward ES. Engineering the Fc Region of Immunoglobulin G to Modulate In Vivo Antibody Levels. Nat Biotechnol (2005) 23(10):1283–8. doi: 10.1038/nbt1143 16186811

[43] Gan Z Ram S Vaccaro C Ober RJ Ward ES. Analyses of the Recycling Receptor, FcRn, in Live Cells Reveal Novel Pathways for Lysosomal Delivery. Traffic (2009) 10(5):600–14. doi: 10.1111/j.1600-0854.2009.00887.x PMC2813311 19192244

[44] Igawa T Haraya K Hattori K. Sweeping Antibody as a Novel Therapeutic Antibody Modality Capable of Eliminating Soluble Antigens From Circulation. Immunol Rev (2016) 270(1):132–51. doi: 10.1111/imr.12392 26864109

[45] Chu SY Horton HM Pong E Leung IW Chen H Nguyen DH. Reduction of Total IgE by Targeted Coengagement of IgE B-Cell Receptor and FcgammaRIIb With Fc-Engineered Antibody. J Allergy Clin Immunol (2012) 129(4):1102–15. doi: 10.1016/j.jaci.2011.11.029 22257644

[46] Vidarsson G Dekkers G Rispens T. IgG Subclasses and Allotypes: From Structure to Effector Functions. Front Immunol (2014) 5:520. doi: 10.3389/fimmu.2014.00520 25368619 PMC4202688

[47] Joshi T Ganesan LP Cao X Tridandapani S. Molecular Analysis of Expression and Function of Hfcgammariibl and B2 Isoforms in Myeloid Cells. Mol Immunol (2006) 43(7):839–50. doi: 10.1016/j.molimm.2005.06.037 16051361

[48] Miettinen HM Rose JK Mellman I. Fc Receptor Isoforms Exhibit Distinct Abilities for Coated Pit Localization as a Result of Cytoplasmic Domain Heterogeneity. Cell (1989) 58(2):317–27. doi: 10.1016/0092-8674(89)90846-5 2568890

[49] Skogh T Blomhoff R Eskild W Berg T. Hepatic Uptake of Circulating IgG Immune Complexes. Immunology (1985) 55(4):585–94. PMC1453780 4018843

[50] Ganesan LP Kim J Wu Y Mohanty S Phillips GS Birmingham DJ. FcgammaRIIb on Liver Sinusoidal Endothelium Clears Small Immune Complexes. J Immunol (2012) 189(10):4981–8. doi: 10.4049/jimmunol.1202017 PMC4381350 23053513

[51] Iwayanagi Y Igawa T Maeda A Haraya K Wada NA Shibahara N. Inhibitory FcgammaRIIb-Mediated Soluble Antigen Clearance From Plasma by a pH-Dependent Antigen-Binding Antibody and Its Enhancement by Fc Engineering. J Immunol (2015) 195(7):3198–205. doi: 10.4049/jimmunol.1401470 PMC4574519 26320252

[52] Devanaboyina SC Khare P Challa DK Ober RJ Ward ES. Engineered Clearing Agents for the Selective Depletion of Antigen-Specific Antibodies. Nat Commun (2017) 8:15314. doi: 10.1038/ncomms15314 28561044 PMC5460014

[53] Sun W Khare P Wang X Challa DK Greenberg BM Ober RJ. Selective Depletion of Antigen-Specific Antibodies for the Treatment of Demyelinating Disease. Mol Ther (2021) 29(3):1312–23. doi: 10.1016/j.ymthe.2020.11.017 PMC7934575 33212299

[54] Kao L Lam V Waldman M Glassock RJ Zhu Q. Identification of the Immunodominant Epitope Region in Phospholipase A2 Receptor-Mediating Autoantibody Binding in Idiopathic Membranous Nephropathy. J Am Soc Nephrol (2015) 26(2):291–301. doi: 10.1681/ASN.2013121315 25205735 PMC4310656

[55] Fresquet M Jowitt TA Gummadova J Collins R O'Cualain R McKenzie EA. Identification of a Major Epitope Recognized by PLA2R Autoantibodies in Primary Membranous Nephropathy. J Am Soc Nephrol (2015) 26(2):302–13. doi: 10.1681/ASN.2014050502 PMC4310666 25288605

[56] Seitz-Polski B Dolla G Payre C Girard CA Polidori J Zorzi K. Epitope Spreading of Autoantibody Response to PLA2R Associates With Poor Prognosis in Membranous Nephropathy. J Am Soc Nephrol (2016) 27(5):1517–33. doi: 10.1681/ASN.2014111061 PMC4849812 26567246

[57] Park JH Riviere I Gonen M Wang X Senechal B Curran KJ. Long-Term Follow-Up of CD19 CAR Therapy in Acute Lymphoblastic Leukemia. N Engl J Med (2018) 378(5):449–59. doi: 10.1056/NEJMoa1709919 PMC6637939 29385376

[58] Maude SL Laetsch TW Buechner J Rives S Boyer M Bittencourt H. Tisagenlecleucel in Children and Young Adults With B-Cell Lymphoblastic Leukemia. N Engl J Med (2018) 378(5):439–48. doi: 10.1056/NEJMoa1709866 PMC5996391 29385370

[59] Schuster SJ Bishop MR Tam CS Waller EK Borchmann P McGuirk JP. Tisagenlecleucel in Adult Relapsed or Refractory Diffuse Large B-Cell Lymphoma. N Engl J Med (2019) 380(1):45–56. doi: 10.1056/NEJMoa1804980 30501490

[60] June CH Sadelain M. Chimeric Antigen Receptor Therapy. N Engl J Med (2018) 379(1):64–73. doi: 10.1056/NEJMra1706169 29972754 PMC7433347

[61] Ellebrecht CT Bhoj VG Nace A Choi EJ Mao X Cho MJ. Reengineering Chimeric Antigen Receptor T Cells for Targeted Therapy of Autoimmune Disease. Science (2016) 353(6295):179–84. doi: 10.1126/science.aaf6756 PMC5343513 27365313

[62] Lee J Lundgren DK Mao X Manfredo-Vieira S Nunez-Cruz S Williams EF. Antigen-Specific B Cell Depletion for Precision Therapy of Mucosal Pemphigus Vulgaris. J Clin Invest (2020) 130(12):6317–24. doi: 10.1172/JCI138416 PMC7685721 32817591

[63] Reinhard L Zahner G Menzel S Koch-Nolte F Stahl RAK Hoxha E. Clinical Relevance of Domain-Specific Phospholipase A2 Receptor 1 Antibody Levels in Patients With Membranous Nephropathy. J Am Soc Nephrol (2020) 31(1):197–207. doi: 10.1681/ASN.2019030273 31843985 PMC6935013

[64] Seifert L Hoxha E Eichhoff AM Zahner G Dehde S Reinhard L. The Most N-Terminal Region of THSD7A Is the Predominant Target for Autoimmunity in THSD7A-Associated Membranous Nephropathy. J Am Soc Nephrol (2018) 29(5):1536–48. doi: 10.1681/ASN.2017070805 PMC5967759 29555830

[65] Stoddard SV Welsh CL Palopoli MM Stoddard SD Aramandla MP Patel RM. Structure and Function Insights Garnered From in Silico Modeling of the Thrombospondin Type-1 Domain-Containing 7A Antigen. Proteins (2019) 87(2):136–45. doi: 10.1002/prot.25640 PMC6876118 30520531

[66] Teachey DT Lacey SF Shaw PA Melenhorst JJ Maude SL Frey N. Identification of Predictive Biomarkers for Cytokine Release Syndrome After Chimeric Antigen Receptor T-Cell Therapy for Acute Lymphoblastic Leukemia. Cancer Discov (2016) 6(6):664–79. doi: 10.1158/2159-8290.CD-16-0040 PMC5448406 27076371

[67] Frey N Porter D. Cytokine Release Syndrome With Chimeric Antigen Receptor T Cell Therapy. Biol Blood Marrow Transplant (2019) 25(4):e123–7. doi: 10.1016/j.bbmt.2018.12.756 30586620

[68] Gust J Hay KA Hanafi LA Li D Myerson D Gonzalez-Cuyar LF. Endothelial Activation and Blood-Brain Barrier Disruption in Neurotoxicity After Adoptive Immunotherapy With CD19 CAR-T Cells. Cancer Discov (2017) 7(12):1404–19. doi: 10.1158/2159-8290.CD-17-0698 PMC5718945 29025771

[69] Tang X Yang L Li Z Nalin AP Dai H Xu T. First-In-Man Clinical Trial of CAR NK-92 Cells: Safety Test of CD33-CAR NK-92 Cells in Patients With Relapsed and Refractory Acute Myeloid Leukemia. Am J Cancer Res (2018) 8(6):1083–9. PMC6048396 30034945

[70] Shimasaki N Jain A Campana D. NK Cells for Cancer Immunotherapy. Nat Rev Drug Discov (2020) 19(3):200–18. doi: 10.1038/s41573-019-0052-1 31907401

[71] Kotanides H Sattler RM Lebron MB Carpenito C Shen J Li J. Characterization of 7A5: A Human CD137 (4-1bb) Receptor Binding Monoclonal Antibody With Differential Agonist Properties That Promotes Antitumor Immunity. Mol Cancer Ther (2020) 19(4):988–98. doi: 10.1158/1535-7163.MCT-19-0893 32241872

[72] Becker PS Suck G Nowakowska P Ullrich E Seifried E Bader P. Selection and Expansion of Natural Killer Cells for NK Cell-Based Immunotherapy. Cancer Immunol Immunother (2016) 65(4):477–84. doi: 10.1007/s00262-016-1792-y PMC4826432 26810567

[73] Marofi F Al-Awad AS Sulaiman Rahman H Markov A Abdelbasset WK Ivanovna Enina Y. CAR-NK Cell: A New Paradigm in Tumor Immunotherapy. Front Oncol (2021) 11:673276. doi: 10.3389/fonc.2021.673276 34178661 PMC8223062

[74] Corthay A. How do Regulatory T Cells Work? Scand J Immunol (2009) 70(4):326–36. doi: 10.1111/j.1365-3083.2009.02308.x PMC2784904 19751267

[75] Zhang AH Yoon J Kim YC Scott DW. Targeting Antigen-Specific B Cells Using Antigen-Expressing Transduced Regulatory T Cells. J Immunol (2018) 201(5):1434–41. doi: 10.4049/jimmunol.1701800 PMC6103823 30021767

[76] Dahan K Debiec H Plaisier E Cachanado M Rousseau A Wakselman L. Rituximab for Severe Membranous Nephropathy: A 6-Month Trial With Extended Follow-Up. J Am Soc Nephrol (2017) 28(1):348–58. doi: 10.1681/ASN.2016040449 PMC5198292 27352623

[77] Tomas NM Meyer-Schwesinger C von Spiegel H Kotb AM Zahner G Hoxha E. A Heterologous Model of Thrombospondin Type 1 Domain-Containing 7a-Associated Membranous Nephropathy. J Am Soc Nephrol (2017) 28(11):3262–77. doi: 10.1681/ASN.2017010030 PMC5661286 28814510

[78] Brandt LJB Barnkob MB Michaels YS Heiselberg J Barington T. Emerging Approaches for Regulation and Control of CAR T Cells: A Mini Review. Front Immunol (2020) 11:326. doi: 10.3389/fimmu.2020.00326 32194561 PMC7062233

